# Wildfire Risk Assessment of Transmission-Line Corridors Based on Naïve Bayes Network and Remote Sensing Data

**DOI:** 10.3390/s21020634

**Published:** 2021-01-18

**Authors:** Weijie Chen, You Zhou, Enze Zhou, Zhun Xiang, Wentao Zhou, Junhan Lu

**Affiliations:** 1 School of Electrical & Information Engineering, Changsha University of Science & Technology, Changsha 410114, China; chenweijie8558@163.com (W.C.); zhouwentao199734@163.com (W.Z.); L18924167973@163.com (J.L.); 2 Electric Power Research Institute, Guangdong Power Grid, Guangzhou 510080, China; zhou_dky@163.com (E.Z.); xiang_dky@163.com (Z.X.)

**Keywords:** wildfire, risk assessment, Naïve bayes, transmission-line corridors

## Abstract

Considering the complexity of the physical model of wildfire occurrence, this paper develops a method to evaluate the wildfire risk of transmission-line corridors based on Naïve Bayes Network (NBN). First, the data of 14 wildfire-related factors including anthropogenic, physiographic, and meteorologic factors, were collected and analyzed. Then, the relief algorithm is used to rank the importance of factors according to their impacts on wildfire occurrence. After eliminating the least important factors in turn, an optimal wildfire risk assessment model for transmission-line corridors was constructed based on the NBN. Finally, this model was carried out and visualized in Guangxi province in southern China. Then a cost function was proposed to further verify the applicability of the wildfire risk distribution map. The fire events monitored by satellites during the first season in 2020 shows that 81.8% of fires fall in high- and very-high-risk regions.

## 1. Introduction

Due to the inhomogeneous distribution of energy resources and power loads in China, a large number of overhead transmission lines pass through forests and mountains to achieve an optimal allocation of power resources [1,2]. Wildfires are prone to occur in transmission-line corridors during the high-incidence periods. If a wildfire occurs under an overhead transmission line, the high temperature and smoke in wildfire would reduce the insulating strength of air gap drastically and induce a trip fault of transmission line. In addition, the reclosing usually fails under wildfire conditions with continuous high temperature, which seriously endangers the reliable operation of power grid [3,4,5].

To improve the wildfire prevention capability of transmission lines, scholars carried out much research, including distribution analysis of widfire occurrence [6,7], transmission-line trip mechanisms caused by wildfire [8,9], fire-spot monitoring algorithms [10,11,12,13] and wildfire risk assessment methods [14,15,16]. Remote sensing satellite is an economical and effective way to monitor wildfires in transmission-line corridors continuously. After identifying the flame strength of wildfire, the tripping risk would be evaluated by comparing the height of fire and transmission line [17,18]. However, this method needs numerous land-surface and environmental parameters to estimate the possible height of wildfire. Once a wildfire occurs near the transmission lines, it may cause a trip within tens of minutes. Furthermore, multiple wildfires usually occur simultaneously during the period of the Spring Festival, Qingming Festival, and autumn harvest in China. It is difficult for operation and maintenance personnel to rush the field and put out the fire in time. Therefore, it is necessary to assess occurrence probability of wildfire to propose differentiated wildfire prevention strategies.

The wildfire risk assessment was initially proposed by forest and fire departments to supporting decisions or policies for general fire, forest management, and fire suppression. Forestry departments in the US and Canada evaluated the large-scale wildfire risk by combining meteorological factors [19,20]. Nevertheless, these meteorology-based assessment methods cannot meet the requirement of spatial accuracy for transmission-line corridors due to the geographical differences. In 2016, State Grid Corporation of China issued a standard of drawing guidelines (DG) for region distribution map of wildfires near overhead transmission lines. In this standard, fire-spot densities and vegetation burning hazard grades are introduced to construct a risk assessment matrix [21]. However, the wildfire occurrence risk is affected by multi-dimension factors [22]. Besides meteorology and vegetation, physiographic and human-related factors are believed to have an important role in affecting wildfire occurrences [23]. Unfortunately, due to the complexity of wildfire occurrence there is still no physical model that could assess wildfire risk with specific variables.

Bayesian network is an effective approach to estimate uncertainty in risk evaluation in terms of the likelihood of risks and hazards [24]. In this paper, we aim to propose a wildfire risk assessment model based on Naïve Bayes Network (NBN) and remote sensing data. The region of Guangxi province which locates in southern China, is selected as the study area. A total of 14 sub-categories of wildfire-related factors including anthropogenic, physiographic, and meteorologic factors are collected. Then the spatial data are divided into grids of 1 km × 1 km to meet the spatial accuracy requirement of power grid. Considering the historical wildfire occurrences, the grids are divided based on whether there have been wildfires. After the importance evaluation by the relief algorithm, an NBN-based model is built with the optimal factor subset to map the wildfire risk distribution of Guangxi province. The wildfire risks are then divided into four levels based on the wildfire occurrence probability. In addition, a cost function is proposed to evaluate the applicability of wildfire risk map.

## 2. Study Area and Data Collection

### 2.1. Study Area

Guangxi province is in South China with the latitude of 20°54′–26°24′ and the longitude of 104°28′–112°04′. The total area is 237,600 square kilometers. It is located on the southeastern edge of the Yunnan–Guizhou Plateau. In addition, the terrain is high in the northwest and low in the southeast. The region of Guangxi province is dominated by subtropical and tropical monsoon climate, in which the precipitation is synchronous with high temperature. With a large forest area, Guangxi is vulnerable to wildfire disasters due to its changeable climate, complex topography, and various vegetation.

### 2.2. Wildfire-Related Factors

#### 2.2.1. Anthropogenic Factors

Based on survey, more than 90% of wildfires are linked directly or indirectly to intentional and unintentional human activities [25]. It mainly includes productive fires such as wasteland and slash burning, and non-productive fires such as smoking in the wild and incensing on the grave [26]. To represent the influence of human activities, five kinds of anthropogenic sub-factors, which are Distance to Roads (DR), Distance to Settlements (DS), Population Density, Gross Domestic Product (GDP) and Historical Fire-Spot Density, are selected.

Human settlements and roads are the main areas of human activities, in where there are more human-caused fires. Population density reflects the regional population aggregation. A high population density generally corresponds to more human activities and consequently high probability of wildfire occurrence [27]. However, this law may be only suitable to explain the fire events in forests and rural areas. In large urbans, the higher population density may cause the less fire occurrence due to lack of fuels [28]. GDP represents the economic status of regions, which affects the human’s fire habits. The data of roads, settlements, population density were downloaded from the website of the Resource and Environment Science and Data Center (RESDE) with a resolution of 1 km × 1 km. In addition, the DR and DS of grids were calculated by using ArcGIS 10.4.

Historical Fire-Spot Density represents the spatial distribution of wildfires in the past few years. It is related to not only human activities but also other meteorological and physiographic factors. The database of historical fire-spots was monitored by polar orbiting meteorological satellites from 2010 to 2019, which is provided by the National Meteorological Center. The fire-spot data during 2010–2014 is used to calculate the Historical Fire-Spot Density of grids, whereas the remains (from 2015 to 2019) are used to train and test the NBN model. To calculate the Historical Fire-Spot Density, the study area is divided into grids of 2.5 km × 2.5 km first. Then the fire-spots from 2010–2014 were allocate into grids based on their longitude and latitude. In addition, the final Historical Fire-Spot Density of grids was obtained by using Kriging interpolation method from the resolution of 2.5 km × 2.5 km into 1 km × 1 km.

#### 2.2.2. Physiographic Factors

The physiographic factors include the land cover and landscape parameters of grids. The land cover factors are consisted of the Land-Usage Type, Vegetation Type, Fuel Load and Normalized Difference Vegetation Index (NDVI), whereas the landscape parameters are average Elevation, Slope, and Aspect of underlying surface.

The vegetations provide the fuel basis for wildfires’ development. Different types of vegetations differ from their burning capacity in fire ignition and spread. Wildfires prone to ignite and spread rapidly at woodlands, shrubberies and meadows [29]. Therefore, the Land-Usage Type and Vegetation Type are taken to describe the flammability of the underlying surface. To construct the NBN model, the Land-Usage Type and Vegetation Type of study area are classified into four levels according to their flammability, which are shown in Table 1 and Table 2, respectively. The Fuel Loading is represented by the drying weight of fuel per unit area, which affects the spread velocity and flame intensity of fires. The NDVI represents the coverage of surface vegetation. It is another representative parameter about fuel contents on the underlying surface.

Complex topography influences not only the distribution of vegetations, but also the spreading behavior of fires directly. The elevation brings differences of temperature and humidity to affect the composition of vegetations. Moreover, human population tends to cluster at the region with low elevation and gentle slopes, which increases the fire activities. In addition, the slope also has a direct impact on the spreading speed of wildfires [30]. The increase of slope leads to the faster surface runoff, which is beneficial to the drying of vegetations. Slope aspect determines the amount of solar radiation, therefore the humidity of atmosphere and vegetations.

The data of Fuel Loading was obtained from the National Meteorological Center. In addition, other land cover factors and landscape parameters were downloaded from RESDE. All the data resolution are 1 km × 1 km.

#### 2.2.3. Meteorologic Factors

Annual Precipitation and Temperature which have great influences on vegetation growth are selected as the meteorologic factors. In the high precipitation regions, the growth of vegetations is flourishing, which provides fuel conditions for wildfires to burn. However, the reduced transpiration in these areas increases the humidity of vegetation and reduces the flammability. The water-holding capacity of soils and air humidity are also increased. The fires’ ignition and spread are therefore restrained. The higher temperature in forests generally benefits plant growth. In addition, the increase of temperature also accelerates the transpiration of vegetations, which promoting the rapid drying of vegetations. The data of Annual Precipitation and Annual Temperature were obtained from the RESDE, with a resolution of 1 km × 1 km.

### 2.3. Sample Preparation and Pre-Processing

The influencing degree of wildfires in transmission-line corridors varies with the distance. Generally, the fire that is 1 km away from the transmission-line is regarded as the highest risk, whereas the fire that is 3 km away is assumed to be no impact on the operation of transmission-line [31]. To meet the spatial accuracy requirement, the study area is divided into 1 km × 1 km grids. Then all the wildfire-related factors are allocated into grids. Specifically, the grids which have monitored fire-spots from 2015–2019 are taken as the fire samples. Considering the ignition, spread, and extinction of wildfire last for several hours, those monitoring fire-spots within 4 h and 3 km are regarded as a same fire-spot. Meanwhile, a same number of the non-fire samples are sampled randomly from the rest grids of 3 km away from the grids of the fire samples.

Compared to processing continuous factors, the Bayesian model has a higher efficiency and better robustness for discrete factors [24]. Therefore, the equal frequency method integrating empirical knowledge was used to discretize the factors. The standards of factor discretization are shown in Table 3.

### 2.4. Spatial Distribution of Factors

**Figure 1:**
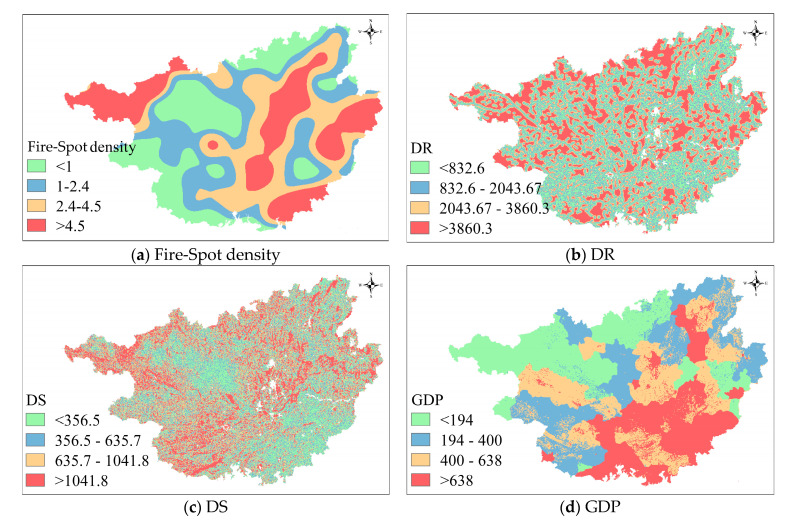

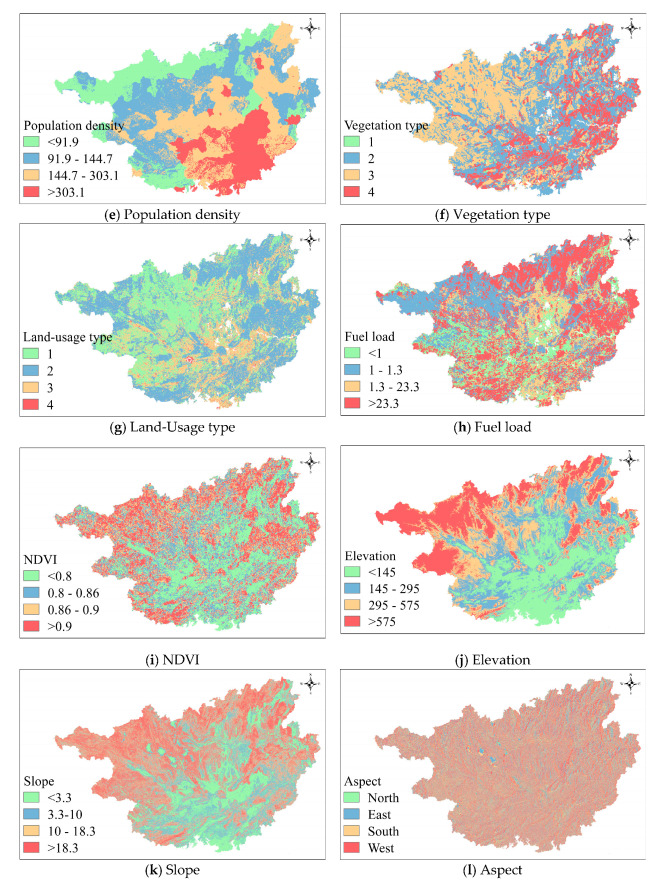

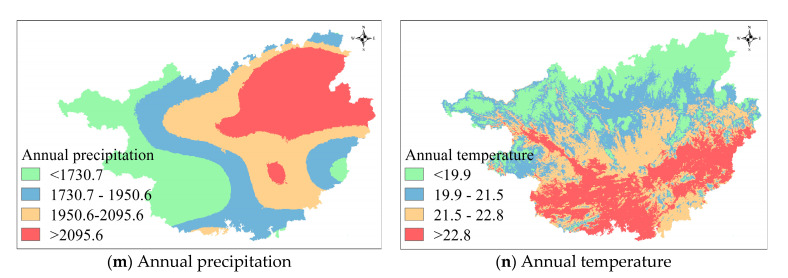
Spatial factor distribution of Guangxi province.

The factor distributions of the study area are visualized by using the ArcGIS software, as shown in Figure 1. It can be seen from the spatial distribution map that the historical wildfire high-incidence areas mainly distribute in the northwest, east, and central parts of Guangxi province. The distribution of GDP is similar to that of population density. The population concentrates in the vicinity of large municipal districts such as Nanning City and Liuzhou City in the central part, and Wuzhou City in the east. The elevation, slope, and NDVI in Guangxi province are relatively similarly distributed. Guangxi province has a wide distribution of karst landforms with mountains and hills. The west and north of Guangxi province are adjacent to the Yunnan–Guizhou Plateau, in where the elevation and slope values are relatively large. As the elevation increases, the climate, hydrothermal conditions change, leading to the plant no longer flourish in these regions. In addition, the higher elevation and steeper slope at the northern of Guangxi province make the less human settlements and higher vegetation coverages and higher NDVI values in this region. For the meteorological distribution, the annual temperature in the northern plateau is slightly lower. That is caused by the increase of latitude and elevation in plateau. The precipitation distribution shows that a circular decreasing trend from the east to the west. In addition, the annual precipitation in the northeast of Guangxi province is the largest. In Guangxi province, sparse forests and shrub forests are the main vegetation and land-Usage Type. In addition, coniferous forests and theropencedrymions mainly locates in the eastern region of Guangxi province, whereas the western region is dominated by broad-leaved forests and shrubs.

## 3. Importance of Wildfire-Related Factors

Wildfire occurrences are influenced by many feature factors. However, the relationship among these factors and wildfire is complex and differs from the location of regions [32]. Some of wildfire-related factors may play an important role in some regions but contribute little on the occurrence probability of wildfires in other regions. In addition, they even introduce noisy information for risk analysis in this region. Moreover, the redundancy of data increases the complexity of model and reduces the evaluation performance. A feature selection method, the relief algorithm, was used to appraise the contribution importance of wildfire-related factors on the wildfire occurrences before model building.

**Figure 2:**
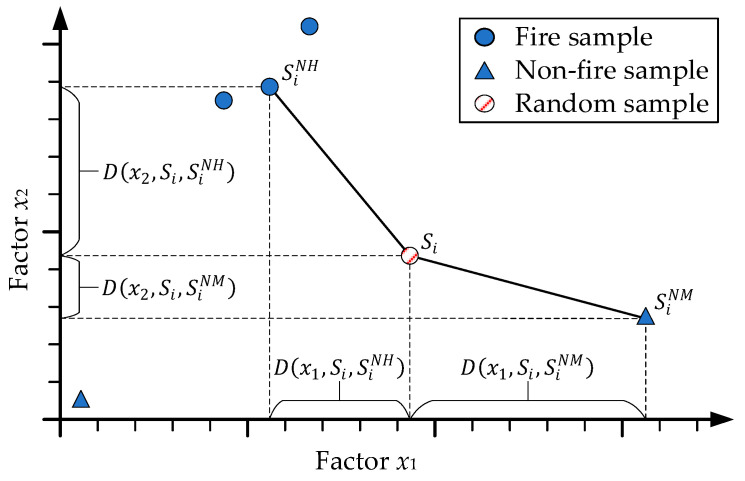
Basic idea of Relief algorithm.

The relief algorithm was first proposed by Kira and Rendell [33], which is a factor weighting algorithm for binary classification based on the correlation between factors and the sample classification. Figure 2 gives the basic idea about how the relief algorithm appraises the importance of two factors. For a random sample $S_{i}$ and its factor $x_{k}$,$D(x_{k},S_{i},S_{i}^{NM})$ represent the distance between and $S_{i}$ and its closest different-class sample $S_{i}^{NM}$. In addition, $D(x_{k},S_{i},S_{i}^{NH})$ represent the distance between $S_{i}$ and its closest same-class sample $S_{i}^{NH}$. If the same-class distance is greater than the different-class distance, the factor is more useful to classify the sample and should be given a higher weight. In Figure 2, if the random $S_{i}$ is a fire sample, then the factor $x_{1}$ has a smaller distance from its same-class samples than that of the factor $x_{2}$, indicating the factor $x_{1}$ is more important than the factor $x_{2}$.

The basic steps of the relief algorithm are as follows:

(1) Take a sample $S_{i}$ randomly from the dataset $D=(S_{1},S_{2},\ldots ,S_{n})$.
(2) Go through the dataset and find the closest same-class sample $S_{i}^{NH}$ and different-class sample $S_{i}^{NM}$ of the $S_{i}^{}$.
(3) Calculate the distance $D(x_{j},S_{i},S_{i}^{NM})$ between the closest samples about a certain factor $x_{j}$. If the factor $x_{j}$ is a discrete variable,
  (1) $$D(x_{j},S_{i},S_{i}^{NM})=\left\{ \begin{array}{l} 0\;\;\;\;\;x_{j}\neq x_{j}^{NM}\\ 1\;\;\;\;\;x_{j}=x_{j}^{NM} \end{array}\right.$$
  else,
  (2) $$D(x_{j},S_{i},S_{i}^{NM})=\left|\frac{x_{j}-x_{j}^{NM}}{\max(x_{j})-\min(x_{j})}\right|$$
  In this study, the most of factors are continuous variables, except the Vegetation Type and Land-Usage Type.
(4) Update the weight of factor $x_{j}$ after multiple sampling
  (3) $$\omega_{j}^{*}=\omega_{j}+\sum \left(\frac{D(x_{j},S_{i},S_{i}^{NM})}{m}-\frac{D(x_{j},S_{i},S_{i}^{NH})}{m}\right)$$
  where $\omega_{j}$ and $\omega_{j}^{*}$ are the initial and updated weight, respectively. m is the number of random sampling. The calculated weights of the 14 wildfire-related factors are listed in Table 4.

Based on relief algorithm, the DS, Vegetation Type, and DR shows the most three important impacts on distinguishing the fire and non-fire samples. This is mainly because of the plentiful of fire activities near the living and transportation regions of populations. In addition, the type of vegetation affects the ignition and spread of wildfires. Population Density, Annual Temperature, Slope, and GDP are the least four important factors. The population in Guangxi province is concentrated in the regions of cities, whereas the rest large parts of region are subtropical forests. In urban area, it is hard to inflame due to the lack of fuels and timely fire-fighting behavior. On the other hand, in sparsely populated forests, the happen probability of wildfire is also low due to the low human activities. The wildfire is likely to be happened at the interfacial region of forests and human settlements, which leads the population and GDP to fade into insignificance in this study. The study area is the provincial power grid in southern of China with a small temperature difference. Thus, the average temperature has little influence on the wildfire occurrence.

## 4. Naïve Bayes Network-Based Wildfire Risk Assessment

### 4.1. Bayes Theorem and Independence Assumption

Bayes’ theorem was initially proposed by Thomas Bayes in the 18th century. It expresses the relationship between the conditional probabilities of two events statistically [34]. It has been widely used in uncertain fields such as disaster prediction, medical diagnosis, speech recognition, and so on. The Bayes’ theorem is as follows:

(4) $$P(X_{i}|Y)=\frac{P(Y|X_{i})\cdot P(X_{i})}{\sum_{j}P(Y|X_{j})\cdot P(X_{j})}$$

where $P(X_{i})$ is the priori probability which are obtained from the past experience or data distribution. $P(Y|X_{i})$ is the probability of event Y occurring under the condition of known event $X_{i}$. $P(X_{i}|Y)$ is the probability of $X_{i}$ when the result Y is known. Based on this theorem, the probabilities of wildfire-related factors and then the wildfire occurrence probability under certain conditions could be estimated statistically. However, the estimation of joint probability $P(Y|X_{i})$ is difficult due to the limited number of samples. Therefore, the Naïve Bayes Network (NBN), in which the factors are assumed to be independent with each other, is used to model the risk of wildfire occurrences. Even through it sacrifices the interaction of factors but still gets an acceptable performance of model in many applications.

### 4.2. Model Construction

**Figure 3:**
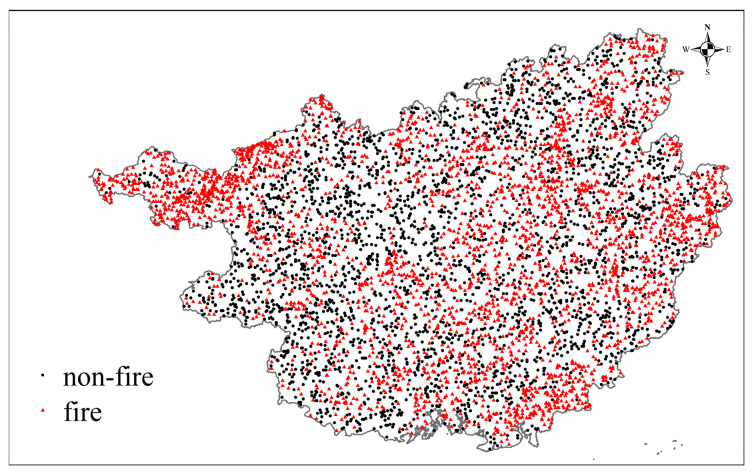
Samples of testing subset in the Guangxi province.

The construction of a Bayes network includes the structure learning and the parameter learning. Due to the independence assumption, the structure of NBN is simplified into a directed acyclic graph with factor nodes connecting to a class node. In addition, the parameter learning process is as follows:

(1) Sample preparation from the grids. The fire samples are the grids where fire-spots have been monitored by satellites in the years from 2015 to 2019. A total of 20,348 fires were recorded. In addition, a same number of non-fire samples were extracted randomly excluding buffer zones of 3 km around the fire samples. Then the samples were graded according to the discretization standards in Table 3. The training subset was randomly chosen from 70% of the samples, the remaining samples were used as the testing subset to evaluate the model’s performance. In addition, the spatial distribution of samples in the testing subset are shown in Figure 3.
(2) By using the training subset, the probabilities of factors are obtained under fire and non-fire condition based on the maximum likelihood estimation.
  (5) $$P(x_{ij}|Y)=\frac{n_{ij}}{\sum_{k=1}^{4}n_{ik}}$$
  where $x_{ij}$ is the *i*th wildfire-related factor fall in *j*th level; and $n_{ij}$ is the number of $x_{ij}$.k represented the discretization level of factors. $P(x_{ij}|Y=0)$ and $P(x_{ij}|Y=1)$ are the probabilities of $x_{ij}$ for the girds with non-fires and fires, separately.
(3) The conditional probability $P(Y=1|x_{1},x_{2},\ldots ,x_{n})$ and $P(Y=0|x_{1},x_{2},\ldots ,x_{n})$ of samples in the testing subset were calculated based on the Bayes’ theorem. In addition, the final wildfire occurrence probability *P(Y)* was obtained after normalization.
  (6) $$P(Y)=\frac{P(Y=1|x_{1},x_{2},\ldots ,x_{n})}{P(Y=1|x_{1},x_{2},\ldots ,x_{n})+P(Y=0|x_{1},x_{2},\ldots ,x_{n})}$$
(4) To test and optimize model’s performance, the value of 0.5 was selected as the threshold to divide the samples into two classes: “Prone to fire” and “Prone to non-fire”. The least important factors were then eliminated in turn to study the influence of factors composition on the model’s performance.

### 4.3. Model Assessment

The confusion matrix is used to evaluate the assessment performance of NBN model. It compares the predicted fire tendency of the testing subset with the actual wildfire events to measure the following four indexes, as shown in Table 5.

*TP*: True Positive, represents the number of fire events correctly predicted as “Prone to fire”.

*TN*: True Negative, indicates the grids where are non-fire and that were identified as “Prone to non-fire”, correctly.

*FN*: False Negative, reflects the fire events that were identified as “Prone to non-fire”, mistakenly.

*FP*: False Positive, recounts the grids where are non-fire but that are predicted to be “Prone to fire”.

It is obviously that larger shares of TP and TN indicate a better predicted performance of model. Therefore, the indexes of *Accuracy P_a_*, $Recall\;P_{r}$, $Precision\;P_{p}$ and a more balanced index F-score are introduced on the basis of the confusion matrix.

(7) $$P_{a}=\frac{TP+TN}{TP+TN+FP+FN}$$

(8) $$P_{r}=\frac{TP}{TP+FN}$$

(9) $$P_{p}=\frac{TP}{TP+FP}$$

(10) $$F=\frac{(1+\beta^{2})P_{p}P_{r}}{\beta^{2}P_{p}+P_{r}}$$

where β reflect the attention degree of power grids on fault tolerance. Considering the wildfire would induce outage of transmission lines, bring huge economic losses, and even casualties, this study takes β as 3.

**Figure 4:**
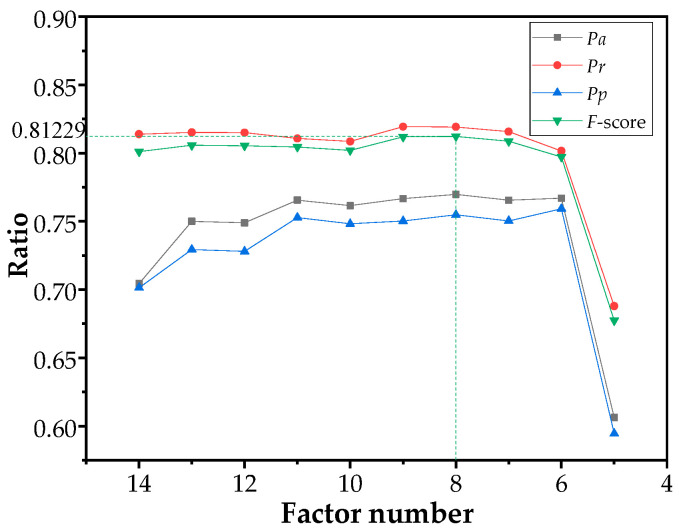
Variation of assessment indexes with wildfire-related factors.

As mentioned in Table 4, the weights of wildfire-related factors have been evaluated by using relief algorithm. Then the NBN models were re-trained and re-tested by eliminating the least important wildfire-related factor one by one. The assessment performances of models are shown in Figure 4.

With the reduced number of wildfire-related factors, the impact of noise, which is bring by unimportant factors, on the model is gradually reduced, which leading to the improvement of $Accuracy\;P_{a}$ and $Precision\;P_{p}$. The $Accuracy\;P_{a}$ is only 70.14% when all 14 wildfire-related factors are used. It reaches the maximum of 75.93% when the number of factors is reduced to six. The $Recall\;P_{r}$ and F-score remain about 81% until the used factor number less than six. However, the performance of NBN model deteriorates significantly when the number of wildfire-related factors reduces from six to five, indicating that the remaining factors have greatest impacts on the wildfire occurrence.

The F-score reaches the highest value of 81.23% when eight wildfire-related factors are used. The results of confusion matrix are listed in Table 6. 81.92% of actual fire events in the testing subset are predicted correctly. The eight wildfire-related factors include three anthropogenic factors (DS, DR, Elevation and Fire-spot density), three physiographic factors (Vegetation Type, land-Usage Type, and NDVI) and only one meteorological factor (Annual Precipitation). The composition of important wildfire-related factors indicates an important role of human activities in increasing the risk of wildfires. Based on survey, more than 90% of wildfires in Guangxi province are human-caused, deliberately and unintentionally.

## 5. Visualization of Wildfire Risk and Discussion

**Figure 5:**
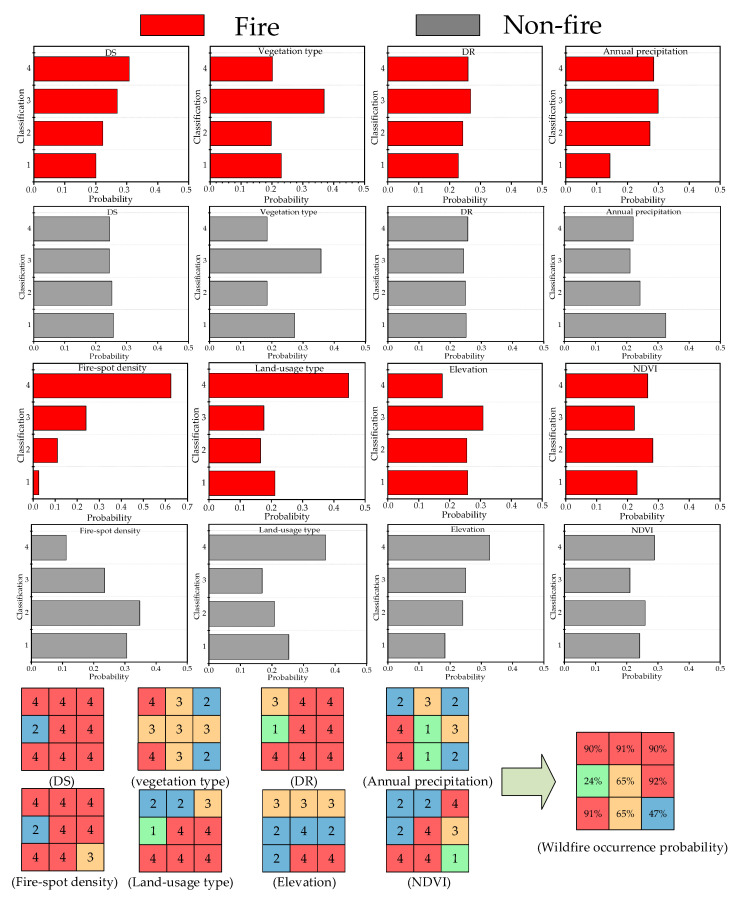
Conditional probability distribution of wildfire-related factors and an example of probability inference.

To guide the wildfire prevention of transmission corridors, the wildfire occurrence probability is calculated in all 1 km × 1 km grids of the study area. The wildfire risks of grids are then divided into four levels based on the probability: Low-(0% ≤ *p* < 25%), Medium-(25% ≤ *p* < 50%), High-(50% ≤ *p* < 75%) and Very-high-(*p* ≥ 75%) risk. The conditional probability distribution of wildfire-related factors and an example of probability inference for grids are shown in Figure 5. The remarkable difference of conditional probability distribution gives a visualized explanation of why and how these wildfire-related factors affect wildfire occurrence.

**Figure 6:**
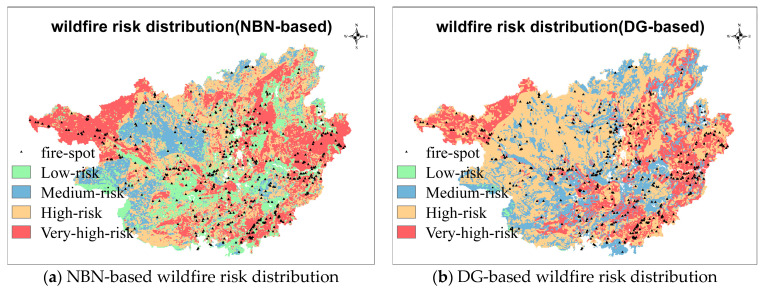
Wildfire risk distribution in Guangxi province.

The NBN-based wildfire risk distribution of Guangxi province is mapped by using the ArcGIS software (Figure 6a). For comparison, another wildfire risk distribution map is drawn according to the DG of State Grid Corporation of China (Figure 6b) [21]. In addition, a total of 527 fire events, which were monitored by remoting satellite during the first season in 2020, allocate on the maps.

It can be observed in the NBN-based map that the very-high-risk regions mainly locate at the northwest, south, and east of Guangxi province, which has a high spatial consistency with the historical fire-spot density distribution. In addition, the northwest of Guangxi province is in the border of Yunnan–Guizhou Plateau. The higher elevation and less annual precipitation result in low moisture in air and vegetation, which is the flammable condition for wildfires. In the eastern and southern regions, the population density is higher, and the settlements are widely distributed, therefore more fire-using activities. In addition, low-risk regions distribute at the middle of Guangxi province, which are generally municipal districts with few fuels and the deep forests without human activities.

The distribution of very-high-risk regions in DG-based map are almost the same as that in NBN-based map, but few low-risk areas are observed in the DG-based map. The area proportions of risk levels, as well as the location proportions of fire events, are summarized in Table 7. Due to the huge ratio of forest in Guangxi province, more areas are assessed as high-and very-high-risk level by both NBN-based map and DG-based map. Compared to NBN-based map, the area proportions of risk levels in DG-based map are more inhomogeneous. More than half of regions in Guangxi province are assessed to be high risk.

Table 7 also gives the proportions of fire events in risk level regions. 45.35% of fire events distribute in the very-high-risk regions of NBN-based map, whereas 32.45% in that of DG-based map, indicating a higher prediction precision of NBN-based map. However, it should be noticed that the higher predicted precision of NBN-based map may be caused by the bigger proportion of very-high-risk region, which are 31.67% and 23.00% for the NBN-based map and DG-based map, respectively. A larger area of high-risk region indicates a greater probability of fire event happening in the region, which leads to the better prediction of model. On the other hand, the larger area of high-risk region needs more costs for wildfire rescue and management. To balance the contradiction of prediction precision and management cost, a cost index -score is proposed.

(11) $$R=\sum_{i=1}^{4}\left(k_{i}\times S_{i}+f_{i}\times N_{i}\right)$$

where $k_{i}$ are the maintenance cost for the region with the ith risk level; The maintenance costs of different risk levels need further studies in power grid cases, and are simplified to 1, 2, 4, 8 for the low-risk, medium-risk, high-risk, and very-high-risk regions, respectively. $S_{i}$ is the area proportion of the ith risk region. In addition, $f_{i}$ represents the misjudgment cost when the wildfires are happened in the ith risk level. If a fire happened in the low-risk and medium-risk region, it is likely to be enlarged and bring disaster cost due to negligence of management. Thus, $f_{i}$ are set as 8, 8, 2, and 0 for simplicity. The R-score of NBN-based map is 6.15, which is lower than that of DG-based map (6.62). The reduced cost also indicates the applicability of NBN-based map.

## 6. Conclusions

This study develops a spatial framework to assess and map wildfire risk of transmission-line corridors by integrating remote sensing data. The proposed NBN-based wildfire risk assessment combines empirical knowledge and machine learning into the discretization of input factors, construction of conditional probabilities of wildfire-related factors, and mapping of wildfire risk distribution. NBN as a core algorithm is used to infer the probabilities of wildfire occurrence. The remote sensing data including a total of 14 sub-categories of wildfire-related factors is assembled to construct NBN model. Based on the relief algorithm, the number of key wildfire-related factors is reduced into 8, indicating human activities and fuels at underlying surface play more important roles in wildfires occurrence. This spatial framework was implemented in a case study of Guangxi province in the south of China. In addition, a cost index, R-score, is proposed to reflect the maintenance costs and misjudgment costs. The results show that the NBN-based wildfire risk map has a higher prediction precision and lower costs for power grids than the traditional method. 45.35% of new monitored fire events distribute at the very-high-risk regions. The visual wildfire risk distribution can assist decision maker of power grids to optimize both supplies and staff resources and make strategies for responding damage control in the future.

## Figures and Tables

**Table 1:** Classification of Vegetation Types.

Level	Description
1	Desert, Swamp, Cultivated plants
2	Meadow, Grassland, Alpine vegetation
3	Broad-leaved forest, Shrub
4	Coniferous forest, theropencedrymion

**Table 2:** Classification of Land-Usage Types.

Level	Description
1	Paddy field, Dry land, Water area, Unused land, Urban-rural fringe, Industrial and mining land, Residential land
2	Meadow, Grassland, Alpine vegetation
3	Broad-leaved forest, Shrub
4	Coniferous forest, Theropencedrymion

**Table 3:** Factor discretization standards.

Factors	Discrete Intervals
GDP (10,000 yuan/km^2^)	(0, 194), (194, 400), (400, 638), (638, ∞)
Fuel load(t/km^2^)	(0, 1), (1, 1.3), (13, 23.3), (23.3, ∞)
NDVI	(0, 0.8), (0.8, 0.86), (0.86, 0.90), (0.90, 1]
Population density (people/km^2^)	(0, 91.9), (91.9, 144.7), (144.7, 303.1), (303.1, ∞)
Elevation (m)	(0, 145), (145, 295), (295, 575), (575, ∞)
Fire-spot density (unit/(100 km^2^·year))	(0, 1), (1, 2.4), (2.4, 4.5), (4.5, ∞)
Annual precipitation (mm)	(0, 173.1), (173.1, 195.1), (195.1, 209.6), (209.6, ∞)
Annual temperature (°C)	(0, 19.9), (19.9, 21.5), (21.5, 22.8), (22.8, ∞)
Slope (°)	(0, 3.3), (3.3, 10), (10, 18.3), (18.3, 90]
Aspect (°)	North (0°, 45°) ∪ (315°, 360°), East (4 5°, 135°), South (135°, 225°), West (225°, 315°)
DS (m)	(0, 356.5), (356.5, 635.7), (635.7, 1041.8), (1041.8, ∞)
DR (m)	(0, 832.6), (832.6, 2043.7), (2043.7, 3860.3), (3860.3, ∞)

**Table 4:** Weights of wildfire-related factors based on the Relief.

Wildfire-Related Factor	Weight
DS	0.1265
Vegetation Type	0.1227
DR	0.1182
Annual precipitation	0.1043
Fire-spot density	0.0997
Land-Usage Type	0.0922
Elevation	0.0873
NDVI	0.0789
Aspect	0.0554
Fuel load	0.0376
Population density	0.0297
Annual temperature	0.0245
Slope	0.0134
GDP	0.0096

**Table 5:** Definition of confusion matrix.

Samples in Testing Subset		Predicted Results	
		Prone to Fire	Prone to Non-fire
Actual events	Fire	TP	FN
	Non-fire	FP	TN

**Table 6:** Results of confusion matrix when 8 wildfire-related factors are used

		Predicted Results		
		Prone to Fire	Prone to Non-fire	Total
Actual events	Fire	4839	1161	6000
	Non-fire	1641	4359	6000
	Total	6480	5520	—
$Accuracy\;P_{a}$		76.65%		
$Recall\;P_{r}$		80.65%		
$Precision\;P_{p}$		74.68%		
F-score		81.23%		

**Table 7:** Comparison of NBN-based map and DG-based map

	NBN-Based Map		DG-Based Map	
	Fire Proportion	Area Proportion	Fire Proportion	Area Proportion
Low-risk	8.92%	19.94%	0.00%	0.28%
Medium-risk	9.30%	16.93%	22.20%	26.40%
High-risk	36.43%	31.46%	45.35%	50.32%
Very-high-risk	45.35%	31.67%	32.45%	23.00%
R-score	6.15		6.62	

## Data Availability

No statement.
